# The CaMKII holoenzyme structure in activation-competent conformations

**DOI:** 10.1038/ncomms15742

**Published:** 2017-06-07

**Authors:** Janette B. Myers, Vincent Zaegel, Steven J. Coultrap, Adam P. Miller, K. Ulrich Bayer, Steve L. Reichow

**Affiliations:** 1 Department of Chemistry, Portland State University, Portland, Oregon 97021, USA; 2 Department of Pharmacology, University of Colorado, Aurora, Colorado 80045, USA

## Abstract

The Ca^2+^/calmodulin-dependent protein kinase II (CaMKII) assembles into large 12-meric holoenzymes, which is thought to enable regulatory processes required for synaptic plasticity underlying learning, memory and cognition. Here we used single particle electron microscopy (EM) to determine a pseudoatomic model of the CaMKIIα holoenzyme in an extended and activation-competent conformation. The holoenzyme is organized by a rigid central hub complex, while positioning of the kinase domains is highly flexible, revealing dynamic holoenzymes ranging from 15–35 nm in diameter. While most kinase domains are ordered independently, ∼20% appear to form dimers and <3% are consistent with a compact conformation. An additional level of plasticity is revealed by a small fraction of bona-fide 14-mers (<4%) that may enable subunit exchange. Biochemical and cellular FRET studies confirm that the extended state of CaMKIIα resolved by EM is the predominant form of the holoenzyme, even under molecular crowding conditions.

## Abstract (web summary)

Ca^2+^/calmodulin-dependent protein kinase II (CaMKII) forms a 12 subunit holoenzyme central to synaptic plasticity. Here the authors report a 3D structure of the CaMKII holoenzyme in an activation-competent state obtained by single particle EM, and suggest a role for the intrinsically disordered linker domain in facilitating cooperative activation.

The Ca^2+^/calmodulin(CaM)-dependent protein kinase II (CaMKII) α isoform is a central mediator of synaptic plasticity that underlies learning, memory and cognition^1^,^2^,^3^. A hallmark feature of CaMKII regulation is autophosphorylation at T286, which generates Ca^2+^/CaM-independent ‘autonomous’ kinase activity that persists even when the initial Ca^2+^-stimulus subsides. T286-phosphorylation has been described as a form of molecular memory, and is indeed required for normal learning and memory, as well as for two opposing forms of synaptic plasticity, long-term potentiation and depression^4^,^5^. Another form of molecular memory by CaMKII is formed through the regulated binding to the NMDA-type glutamate receptor subunit GluN2B, which can also generate autonomous activity^6^. This CaMKII/GluN2B binding is specifically required for maintenance of synaptic strength^7^ and for normal long-term potentiation but not long-term depression^8^.

Both forms of molecular memory are enabled by the CaMKII holoenzyme structure, which is assembled by the association of 12 subunits via their C-terminal association domains (also termed hub domains). GluN2B binding is mediated by the kinase domain, but is dramatically more efficient for holoenzymes compared with monomers^6^,^9^,^10^. T286-autophosphorylation occurs as an inter-subunit reaction within the holoenzyme, by a mechanism that enables frequency-detection by CaMKII (refs 11, 12, 13). Additionally, holoenzyme formation is thought to enable structural functions of CaMKII (refs 14, 15, 16).

Several crystal structures of individual CaMKII domains have been described^17^,^18^,^19^,^20^,^21^,^22^. However, the structure of the holoenzyme assembly that determines the relative positioning of the kinase domains to each other remains elusive. The principle domain sequence of a CaMKII subunit is illustrated in (Fig. 1a), with an N-terminal kinase domain, followed by a short regulatory domain that contains T286, the variable linker domain that is subject to alternative splicing, and the C-terminal hub domain. A recent crystallographic model has been described for an artificial CaMKIIα construct in which the variable linker domain was completely deleted (linker-less)^23^. After bacterial expression, this linker-less construct formed a compact conformation, in which the kinase/regulatory domains are packed closely against the central hub complex. However, in this compact conformation, the regulatory domains are inaccessible to Ca^2+^/CaM-stimulation. Thus, in order to make the holoenzyme competent for activation by Ca^2+^/CaM, it has been proposed that at least one alternative extended conformation must exist, even under basal-state conditions^1^,^18^,^23^. Additionally, in order to enable the inter-subunit autophosphorylation at T286, there must be a conformation with a flexible kinase domain positioning that allows for one kinase domain to access the regulatory domain of its neighbour. Such flexibility could either be induced by Ca^2+^/CaM-stimulation or be an intrinsic property of the activation-competent conformation of a holoenzyme that is present even in its basal state.

For the compact CaMKII conformation, the biological occurrence and implications remains to be elucidated, especially in context of a naturally occurring full-length CaMKII that contains the linker region. However, it has been suggested that an equilibrium between the compact conformation and an additional activation-competent conformation would shape the CaMKII activation characteristics: it would make the stimulation by Ca^2+^/CaM cooperative and thereby also fine-tune the frequency-dependent response of CaMKII to Ca^2+^-oscillation^23^. However, an alternative mechanism for the cooperativity of Ca^2+^/CaM-stimulation has also been suggested. This alternative model is based on a previous crystal structure of isolated kinase/regulatory domains in which two kinase subunits dimerized via a coiled-coil interaction formed by their Ca^2+^/CaM-binding regulatory domains^19^. Thus, it is proposed that dimerization would prevent direct activation of this form as well. However, binding of CaM to one of these paired subunits would facilitate subsequent cooperative CaM-binding to the second subunit that was part of the dimer pair^19^. While the compact holoenzyme conformation and the kinase domain dimers cannot occur at the same time, both could be part of an equilibrium exchange. However, any equilibrium must include at least one additional activation-competent conformation in which the Ca^2+^/CaM-binding sites are accessible for holoenzyme activation.

Here we set out to investigate the CaMKIIα holoenzyme structure and potential conformational equilibrium using a combination of single particle electron microscopy (EM) and functional mutation studies. Briefly, we describe a three-dimensional (3D) EM structure of an activation-competent CaMKIIα holoenzyme in an extended-state conformation at pseudo-atomic resolution. Single particle analysis revealed a high degree of flexibility for kinase domain positioning, with almost none of the individual particles completely matching the average structure. Within this continuum of conformational states, a compact conformation was found for <3% of the individual kinase domains, but various dimer-pair arrangements were suggested for as much as ∼20% of the kinase domains. Biochemical and cellular studies indicate that the extended state structures described by EM predominate even under conditions of molecular crowding, and point to an important role of the intrinsically disordered linker domain in facilitating cooperative activation for wild type holoenzymes.

## Results

### An extended activatable state of CaMKIIα revealed by EM

**Figure 1:**
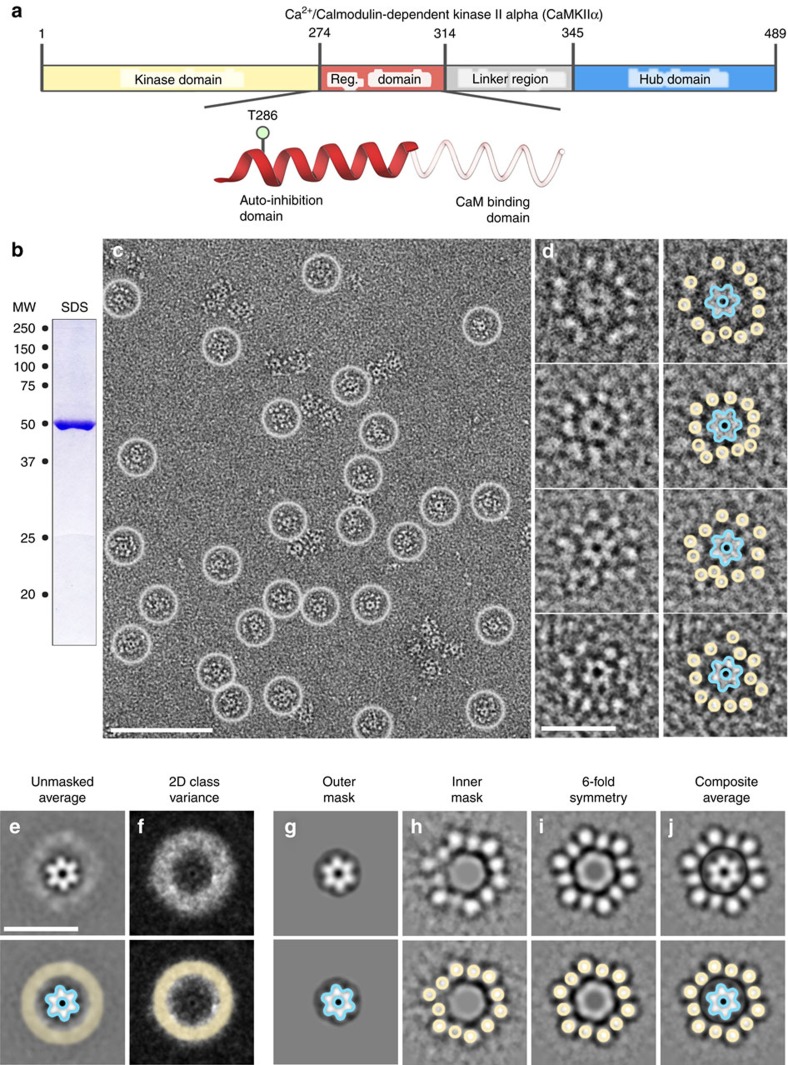
An extended form of the CaMKIIα holoenzyme resolved by single particle EM. (**a**) Diagram of the CaMKIIα domain architecture (numbering of human isoform). Inset, shows a secondary structure diagram of the CaMKIIα regulatory domain. The coil region (faded red) represents a region that is disordered in the absence of calmodulin^21^. The site of auto-phosphorylation (T286) involved in regulating autonomous activity is indicated. (**b**) SDS–polyacrylamide gel electrophoresis of purified human CaMKIIα stained with Coomassie blue migrating at the expected molecular weight (MW ∼50 kDa). (**c**) Electron micrograph of negatively stained CaMKIIα particles. Contrast of protein is white on a dark background. Individual particles are indicated by white circles. Scale bar=100 nm. (**d**) Enlarged view of individual particles with the hub complex (blue outline) and twelve kinase domains (yellow circle) indicated. Scale bar=25 nm. (**e**,**f**) Single particle averaging and image analysis. Scale bar=25 nm. (**e**) Representative 2D projection average of unmasked particles. The central hub complex is clearly defined (blue outline), while the radial kinase domains appear as a diffuse halo (yellow circle). (**f**) 2D variance map of data in **e**. A region of high variance (*white pixels*) corresponds with the blurred region observed in 2D class averages (yellow circle). (**g**) Representative 2D projection average using an applied image mask (75 Å outer radius). The sixfold symmetric hub complex is clearly defined (blue outline). (**h**) Representative 2D projection average with an applied mask to remove contribution of the hub domain during the alignment procedure (50 Å inner radius mask). Twelve kinase domains are visualized at an approximate radius of 24–28 nm (yellow circles). (**i**) Class average in **h** with applied sixfold rotational averaging indicates a pseudo-symmetric kinase domain organization. (**j**) Composite image of **g** and **i** with the identified hub complex (blue outline) and kinase domains (yellow outline) indicated.

CaMKII holoenzymes were prepared for EM using negative stain (Fig. 1). This procedure enabled high-contrast single particle image analysis, which turned out to be important for assessing the extreme structural heterogeneity in holoenzyme architecture determined here. In raw micrographs, individual particles appear as ‘flower-like’ shapes with a defined central ring of protein density surrounded by an array of smaller densities, ‘petals’ (Fig. 1c,d). The central ring of density appears as a six-pointed star (diameter=∼110 Å) with a distinct central pore (diameter=∼25 Å) (Fig. 1d, blue outline). Surrounding the ring structure, up to twelve punctate densities are observed at an approximate diameter of ∼24–28 nm (Fig. 1d, yellow circles). These peripheral features correspond to the twelve kinase domains, assembled by the dodecameric hub complex^24^. The central hub was found to adopt a single highly preferred orientation; by contrast, the configuration of the radial kinase domain densities appeared highly variable with respect to the central hub feature (Fig. 1c,d). These general features were consistently observed with a variety of negative stain reagents and in vitrified specimens (Supplementary Figs 1 and 2).

Two-dimensional classification and projection averaging produced highly populated classes with well-defined hub assemblies and clear sixfold symmetry. However, the peripheral kinase densities were not resolved in these most populated classes. Rather, these domains appeared as a halo of density surrounding the central hub (Fig. 1e). The halo feature corresponds to a region of high variance in these two-dimensional (2D) class averages (Fig. 1f). Inspection of the individual particles within these classes confirmed that this diffuse signal is produced by the variable arrangement of kinase domains. Separating this ∼10,000 particle image data set into a larger number of 2D class averages (for example, >200–500 classes) was only partially successful at resolving holoenzyme structures. The resulting distribution of class averages displayed either resolved hub domains and unresolved kinase domains, or partially resolved kinase domains and unresolved hub domains (Fig. 1e and Supplementary Figs 1 and 2). These results were considered indicative of a central hub domain complex that is connected to twelve peripheral kinase domains by disorganized or flexible polypeptide chains, thus preventing their co-alignment. Therefore, alternative 2D classification approaches were explored to deconvolute the structural arrangement(s) of the holoenzyme, by focusing on each of these domains separately. The results are described below.

Focused 2D classification of the hub domain was performed using a circular image mask (outer radius=75 Å). The vast majority of these masked particles classified as sixfold symmetric structures, with dimensions matching the crystallized dodecameric hub domain assembly^21^,^22^,^23^ (Fig. 1g and Supplementary Fig. 3). In the next round of focused 2D classification, a new image mask was applied to remove the density representing the central hub complex (50 Å inner radius). This approach now clearly resolved up to twelve individual kinase domain densities, arranged radially along a diameter of ∼24–28 nm (Fig. 1h and Supplementary Fig. 4). In a few classes, an apparent pseudo-symmetric arrangement of kinase densities was observed (as in Fig. 1h), and supported by rotational averaging (Fig. 1i). A composite of Fig. 1g,h provides an enhanced view of CaMKIIα holoenzymes observed in raw micrographs, represented by a dodecameric hub complex surrounded by an array of twelve independently arranged kinase domains (Fig. 1j). In addition to this representative average structure, a variety of other kinase domain arrangements (including asymmetric forms) where also observed in single particle images and in 2D class averages (Fig. 1d and Supplementary Fig. 4). Here and in the following section, we have focused on structures where all twelve kinase domains are clearly resolved. In later sections, we describe additional alternative conformational forms that were identified for the basal-state holoenzyme in more detail.

### Pseudo-atomic model of CaMKIIα in a fully activatable state

**Figure 2:**
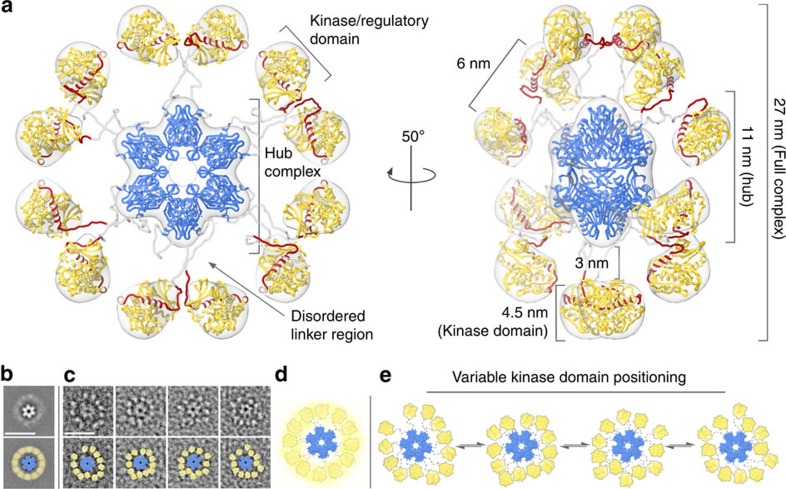
3D-reconstruction and pseudo-atomic model of the CaMKIIα holoenzyme. (**a**) 3D-reconstruction of the CaMKIIα holoenzyme (grey transparent) refined to ∼20 Å resolution. A pseudo-atomic model of the dodecameric holoenzyme (coloured as in Fig. 1a) was constructed using previously determined crystal structures corresponding to the dodecameric CaMKIIα hub complex (blue ribbon, PDBID 5IG3 (ref. 22); residues 345–472) and isolated kinase/regulatory domain (yellow/red ribbon, PDBID 2VZ6 (ref. 21); residues 13–300). Residues 301–344 were modelled as disordered linkers connecting each kinase domain to the nearest hub domain. Residues 274–314 correspond to the regulatory domain (red), containing a proximal auto-inhibitory segment and distal calmodulin-binding site. (**b**,**c**) Unmasked 2D class average and single particle images (as shown in Fig. 1) with crystal structures of the hub complex (blue surface) and kinase/regulatory domain (yellow surface) fit into the EM densities. A yellow halo in **b** represents the diffuse positioning of kinase domains in the 2D class average. Scale bars=25 nm. (**d**,**e**) Enlarged view of the fit domains in **b**,**c** illustrating the proposed structural equilibrium and variable kinase domain arrangements observed in single particle images. Flexible linkers connecting individual kinase domains to the central hub complex are represented as grey dotted lines.

A 3D EM density map was obtained at 20 Å resolution by using a combination of tilted-series tomographic imaging and 3D masking routines (Fig. 2a and Supplementary Movie 1). A pseudo-atomic model of the CaMKIIα holoenzyme was constructed by fitting Protein Data Bank (PDB) coordinates for the dodecameric hub complex (PDBID 5IG3 (ref. 22), blue ribbon) and isolated kinase/regulatory domain (PDBID 2VZ6 (ref. 21), yellow and red ribbon) into the EM density map (see Methods and Supplementary Fig. 5). The central density of the EM map is well defined by the crystal structure of the dodecameric hub complex, formed by residues 345–472 (cross-correlation=0.95 at 20 Å resolution). The structured N-terminal kinase/regulatory domain, containing residues 13–300, was best fit computationally into each of the radial densities with the regulatory domain (red ribbon in Fig. 2a) oriented towards the central hub complex (cross-correlation=0.98 at 20 Å resolution). Neighbouring kinase domains were fit separately to reflect the dihedral symmetry imposed by the hub complex and 3D refinement.

The model of the holoenzyme was completed by connecting the structured kinase/regulatory domains to the central hub complex by flexible linkers^25^ (Supplementary Fig. 5). The modelled linkers are not resolved by the EM map, and were, therefore, inferred by the following substantiating evidence. The crystallized construct of the human CaMKIIα kinase/regulatory domain used for our model included the entire regulatory domain (residues 274–314, Fig. 1a). However, residues 301–314 (containing the distal region of the CaM-binding site) were found to be disordered in the crystal structure^21^. The distal region of the CaM-binding site has been shown to be unstructured by solution state EPR studies^26^ and crystallographic analysis of the human β, γ and δ isoforms as well^21^. Primary sequence analysis further indicates residues 315–344 comprising the CaMKIIα variable linker region are intrinsically disordered^27^. Therefore, for the basal-state holoenzyme structure determined here, each N-terminal kinase/regulatory domain was covalently connected to the nearest C-terminal hub domain by a random coil peptide chain, containing residues 301–344 (Fig. 2a, red and grey coil).

The pseudo-atomic model presented here provides a representative average view of the CaMKIIα holoenzyme in an extended (and activatable) conformation. In this form of the holoenzyme, the kinase domains are arranged independently from each other and removed from the central hub complex. The model implies that each kinase domain is tethered to the central hub complex by a flexible chain of amino acids (residues 301–344). Each kinase domain is separated from the central hub complex, extending the radius of the model to ∼135 Å. Neighbouring kinase domains are separated from each other by ∼60 Å (center-to-center distance). The co-planar arrangement of kinase domains is consistent with previous SAXS analysis^19^,^23^. However, in the EM model, each of the kinase/regulatory domains are displaced from the hub complex by ∼30 Å (edge-to-edge) separation (Fig. 2a). In this extended form of the holoenzyme, the CaM-binding sites^21^ (residues 294–314, red ribbon/coil) are solvent exposed and thus positioned for activation by the Ca^2+^/CaM-stimulus. Therefore, the described structure represents an activation-competent state of the CaMKII holoenzyme.

### CaMKIIα forms a continuum of activation-competent structures

Based on single particle image analysis and 2D classification it is apparent that the flexible linker regions facilitate the formation of a variety of other conformational states that do not completely match the averaged 3D reconstruction. This conformational variability is appreciated when crystal structures of individual domains are fitted into the densities of individual particles images (Fig. 2c,e). To quantitate the variable CaMKIIα domain architecture, we conducted a series of measurements and statistical analysis on individual particle images obtained from raw micrographs. This methodology was facilitated by the high contrast provided by the negative staining technique and preferred orientation of particles on the EM grid.

**Figure 3:**
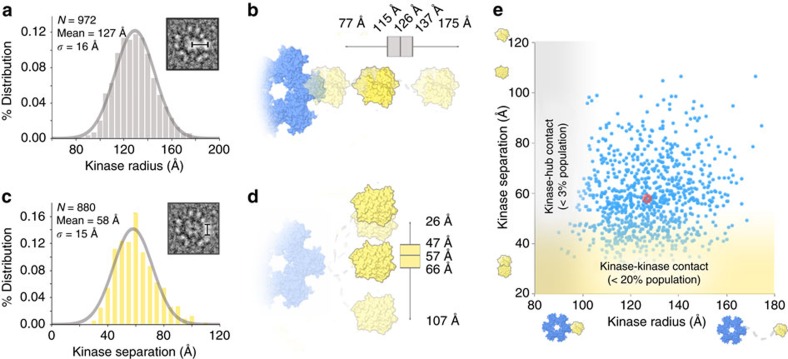
Structural diversity of CaMKIIα kinase domain arrangements measured from single particle images. (**a**) Histogram of measured radius for individual kinase domains. A Gaussian curve fit to the histogram distribution is shown (grey line). (**b**) Illustration and whisker plot representation of the distribution of kinase radius measurements in **a**. Kinase domains (yellow) are illustrated to represent the minimum, maximum and average distance from the hub domain complex (blue). (**c**) Histogram of measured distance separating neighbouring kinase domains (center-to-center). A Gaussian curve fit to the histogram distribution is shown (grey line). (**d**) Illustration and whisker plot representation of the distribution of kinase separation measurements in **c**. Kinase domains are illustrated to represent the minimum, maximum and average kinase separation distances. Inset, in **a**,**c** illustrate the distance measurement made using raw particle images. Whiskers in **b**,**d** indicate the minimum and maximum values, and boxes indicate the 25%, 50% and 75% values of the distribution. (**e**) Correlation map of kinase radius versus kinase separation distance. This analysis revealed no statistical correlation between these values (correlation coefficient=0.2). The average of the two parameters is shown in red. Grey and yellow shading indicates regions of the correlation map where kinase domain positioning is consistent with steric contact with the hub domain and/or kinase-kinase contact, respectively.

In the first set of measurements, the radial extension for each kinase domain (that is, kinase radius) was defined from the center of the hub complex to the outer radius of each kinase domain (Fig. 3a, inset). A histogram obtained from 927 measurements shows a Gaussian distribution of kinase domain radii, with a mean value of ∼127 Å (±16 Å s.d.) (Fig. 3a). This data is consistent with the particle radius obtained by 3D reconstruction (∼135 Å). As the flexible linker region is not directly visible by EM, the extension of the linker itself was estimated by accounting for the volume occupied by the ordered hub complex (∼55 Å radius) and kinase/regulatory domains (∼45 Å diameter). These calculations show that the linker connecting these structured domains adopts an average extension of ∼27 Å, which compares well to the ∼30 Å (edge-to-edge) separation of hub and kinase domain densities in our averaged 3D reconstruction. Theoretical considerations based on random walk theory predict an average extension provided by a 45 residue random coil chain to be ∼24 Å (3.5 Å x ✓N_residues_^28^). The Gaussian distribution of measured radii, and correspondence to the expected theoretical value of a random chain model further support the notion that each kinase domain is freely tethered to the hub complex by an intrinsically flexible linker. In this context, each CaMKII subunit is able to independently sample a continuum of conformations that provide kinase domain extensions ranging from compact forms (minimum radius=77 Å) to highly extended forms (maximum radius=175 Å) (Fig. 3b).

### A fully compact CaMKIIα architecture is not observed by EM

A prominent model of a linker-less CaMKII construct has been determined by X-ray crystallography, revealing a highly compact structure where all twelve kinase domains are packed tightly against the central hub complex^23^. As this compact structure is proposed to have a role in the cooperative activation mechanism of CaMKII, we attempted to recover this form of the holoenzyme using our focused 2D classification methods (using an outer mask radius=110 Å). However, this approach did not identify any structures consistent with 2D back-projections of the linker-less crystal form (Supplementary Fig. 3). This result was corroborated by our statistical analysis performed on individual particle images. A particle radius less than ∼100 Å would potentially place kinase domains in steric contact with the central hub complex. Statistical analysis identified only a small fraction of individual subunits with kinase domain radii that fall within this category (<3% of kinase domains with radius<100 Å) (Fig. 3e). Altogether, these data indicate that an interaction between the hub and kinase domain may only occur for a minor fraction of individual subunits in full-length CaMKIIα holoenzymes, at least under the conditions required for EM.

### Evidence for meta-stable CaMKII kinase domain dimerization

A second model of cooperative activation of CaMKII has been proposed based on the observed dimeric-pairing in the crystal structure of isolated CaMKII kinase domains obtained from *C. elegans*^19^. We assessed the formation of potential kinase domain dimers by measuring the center-to-center distance separating neighbouring kinase domains within our individual particle data set (Fig. 3c, *inset*). This analysis yielded a Gaussian distribution of kinase domain separation distances, centered at ∼58 Å (±15 Å s.d.) (Fig. 3c). This value is remarkably similar to average separation distances determined by previous Förster resonance energy transfer (FRET) studies^29^. Here we additionally considered the approximate volume occupied by each kinase/regulatory domain (∼45 Å diameter) to indicate that the majority of kinase domains are arranged independently (non-interacting) from their neighbouring subunits, as represented by the activation-competent structure determined by EM (Fig. 2a).

In addition to these independent states, a significant population of neighbouring kinase domains (∼20% of the population) were found separated by <45 Å (that is, within steric contact distance) (Fig. 3d,e). This close proximity of neighbouring kinase domains is not simply due to positioning by shorter linker extensions. A distribution plot of kinase domain radius versus kinase domain separation revealed no significant correlation between these values (correlation coefficient=0.2) (Fig. 3e). For example, a kinase domain with an extended radius (for example,>125 Å) may be found closely positioned <45 Å to a neighbouring domain. Vice versa, a kinase domain with a shorter radius (for example,<125 Å) may be separated by >100 Å from one of its neighbouring kinase domains. Altogether these data suggest neighbouring kinase domains may exist in two population states, as isolated independent domains and as meta-stable dimeric pairs.

**Figure 4:**
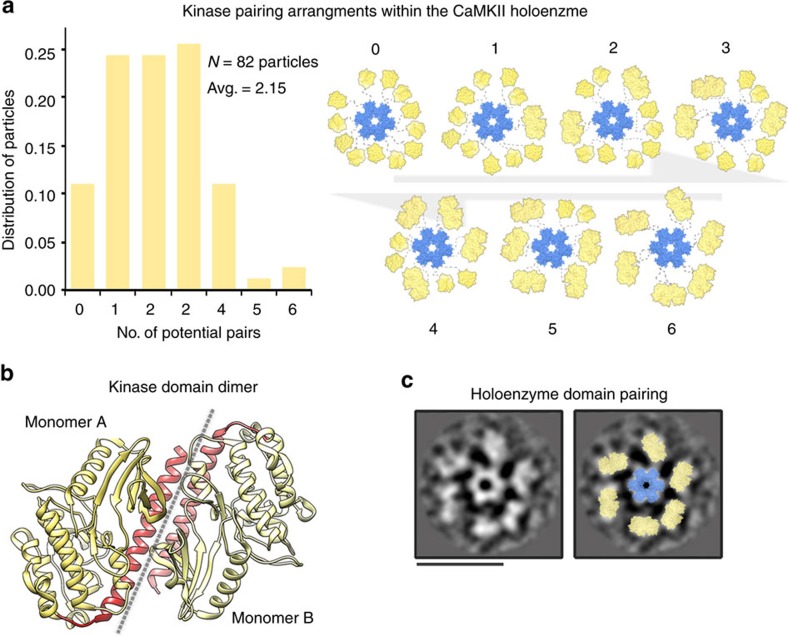
Putative kinase domain pairing in the CaMKIIα holoenzyme observed by single particle EM. (**a**) Left, Distribution of kinase domain pairing arrangements, based on measured inter-domain distances (as described in main text). Holoenzyme structures were categorized as being arranged with 0–6 kinase domain pairs. Right, Illustration representing holoenzyme structures with various kinase domain pairing arrangements. These structures (and other various arrangements of kinase pairing) are suggested to be in equilibrium with various unpaired kinase arrangements. (**b**) Crystallographic structure of the *C*. *elegans* CaMKIIα kinase/regulatory domain (yellow/red ribbon; PDBID 2BDW (ref. 19)) previously shown to form a dimeric interface involving the regulatory domain (*red*). (**c**) *Left,* Reference-free 2D class average observed for a small population of CaMKIIα particles apparently organized with all kinase domains forming paired interactions. *Right,* Crystal structures of the hub complex (blue surface) and dimeric kinase domains (yellow surface) are fit into the EM densities. Scale bar=25 nm.

To assess the various patterns of kinase domain pairing, we categorized the holoenzymes from our same individual image data set according to the number of putative kinase domain pairs, defined as having neighbouring kinase domains separated by <45 Å (Fig. 4a). This analysis identified a variety of arrangements, consisting of 0–6 sets of kinase domain pairs per particle. Approximately 10% of the particles were classified as having all kinase domains unpaired and independently arranged by the holoenzyme (as shown in Figs 1 and 2). Holoenzyme structures with 1–3 kinase domain pairs appeared to be most common (representing a combined ∼60% of the population), while configurations with all twelve kinase domains arranged as dimers appeared to be rare (represented only ∼2.5% of the population).

These individual particle statistics are in agreement with the results obtained by reference-free 2D classification procedures, where a majority of particles classified into structures where only 9–11 peripheral kinase densities are resolved (Supplementary Figs 1 and 4). Although rare, we were able to capture a structure from 2D classification results that is consistent with an arrangement of the holoenzyme where all twelve kinase domains are arranged as kinase dimer pairs (Fig. 4b,c). The crystal structure of *C. elegans* CaMKII kinase domain dimer fits well into the peripheral EM densities of this particular 2D class average (Fig. 4c, right). The low population of particles fitting to this individual class (< 1% of classified particles) is consistent with the determined population distribution in Fig. 4a.

### Wild-type CaMKIIα remains extended under molecular crowding

**Figure 5:**
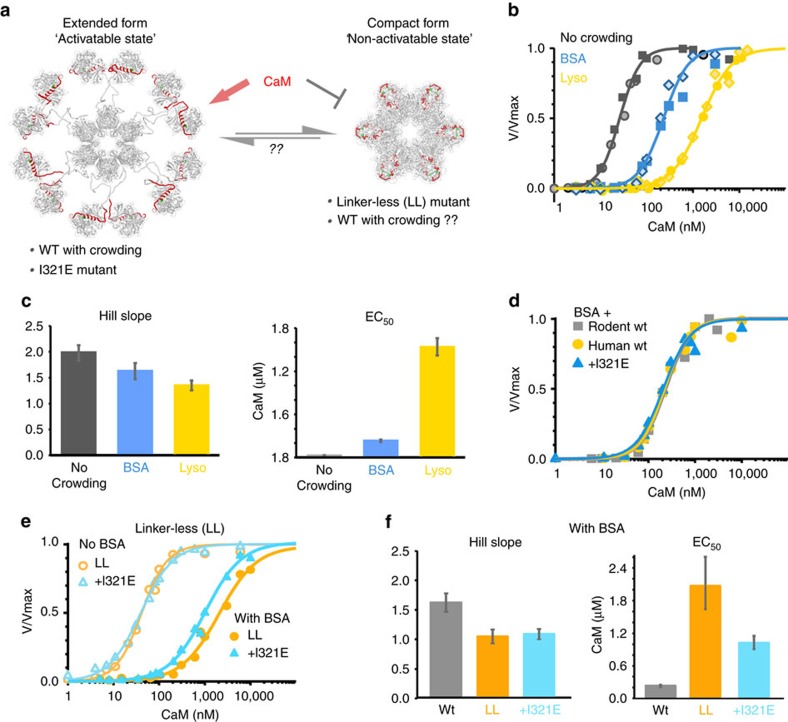
Full-length CaMKIIα wild type expressed in eukaryotic cells remains largely in an extended conformation even during molecular crowding. (**a**) Models of the compact versus extended CaMKII holoenzyme conformations, which differ in their accessibility to Ca^2+^/CaM. The compact conformation has been described for a linker-less (LL) CaMKII and suggested during molecular crowding also for wild type (WT) based on increased Hill slope; the I321E mutation prevented the compact conformation even for the LL mutant^23^. (**b**) Ca^2+^/CaM dose/response curves for *in vitro* activation of rodent CaMKII without crowding (Contr) or with crowding by 150 mg ml^−1^ BSA or lysozyme (lys). The curve fits shown are based on data from two independent experiments; the curve fits from the individual experiments are shown Supplementary Fig. 6. (**c**) The Hill slope was not increased by molecular crowding and thus gave no indication for induction of a compact conformation. By contrast, the EC_50_ was increased dramatically by lysozyme and to a lesser extent by BSA. (**d**) Human CaMKIIα and its I321E mutant that is incompetent for the compact conformation showed the same Ca^2+^/CaM responses during crowding with BSA as rodent CaMKIIα. (**e**,**f**) Linker-less (LL) CaMKII and its I321E mutant showed the same Ca^2+^/CaM response without crowding. However, crowding with BSA caused a lesser increase in E_50_ for the I321E mutant that is incompetent for the compact conformation. The Hill slope was identical for both linker-less mutants and no longer showed significant cooperativity under crowding conditions. Error bars represent the s.e. calculated from the curve fits.

The compact conformation CaMKII holoenzyme model is based on the crystal structure of a linker-less (LL) CaMKIIα mutant expressed in bacteria^23^ (Fig. 5a). This compact form of CaMKII is expected to be a non-activatable state because the CaM-binding site is buried within the compact kinase/hub interface. However, it was suggested that this compact form enables cooperativity, because CaM binding at one subunit would disrupt the compact conformation at adjacent subunits, thereby facilitating subsequent CaM binding to the neighbouring kinase domains^23^. In support of this notion, the (LL) mutant was reported to have a dramatically increased EC_50_ for activation by Ca^2+^/CaM (∼500-fold) and a mildly increased Hill coefficient (∼1.5-fold) compared with full-length wild-type CaMKIIα, consistent with a compact conformation that decreases Ca^2+^/CaM binding and enhances cooperativity in solution^23^. By contrast, our EM studies conducted on diluted full-length CaMKIIα wild type was observed to form a predominant extended conformation, consistent with the <3% of subunits in a potentially compact conformation. However, it was suggested in the previous study that molecular crowding conditions, as found within cells, was required to induce a compact conformation for the wild type full-length CaMKIIα^23^. The evidence for this came from molecular crowding experiments conducted with lysozyme, which increased the Hill coefficient for full-length CaMKIIα wild type by 1.3–2-fold, but not for full-length CaMKII containing an I321E mutation shown to disrupt the compact conformation. Under these molecular crowding conditions, the EC_50_ was reported to increase equally by ∼3-fold for both wild type and I321E mutant, which was interpreted to indicate that the increase in the Hill coefficient (but not in the EC_50_) can be used as a readout of the compact conformation^23^.

A caveat to this previous study, however, is that lysozyme has been shown to bind directly to Ca^2+^/CaM^30^, and, therefore, potentially complicates the interpretation of these results, especially regarding effects on the apparent EC_50_. Thus, we first compared the effects of molecular crowding by 150 mg ml^−1^ lysozyme and the putatively more inert molecule bovine serum albumin (BSA) (Fig. 5b,c and Supplementary Fig. 6). Consistent with lysozyme competition for Ca^2+^/CaM, crowding experiments performed with this reagent dramatically increased the EC_50_, while crowding with BSA caused a significantly smaller effect (Fig. 5c). However, neither crowding condition increased the Hill coefficient; if any, it was slightly reduced (Fig. 5c). Thus, for full-length rodent CaMKIIα wild type expressed in eukaryotic cells, the Hill coefficients in these *in vitro* crowding experiments did not provide any indication for a compact conformation.

Our initial experiments were conducted using rodent CaMKIIα, as compared with the previous study that characterized the human isoform. Rodent and human CaMKIIα differ only in one amino-acid residue (asparagine versus serine at position 324 within the linker region). Nonetheless, we decided to test if this one amino-acid difference could explain the different effect of molecular crowding on the Hill coefficient seen in our study with rodent CaMKII. However, the effects of molecular crowding with BSA on rodent versus human CaMKIIα were indistinguishable (Fig. 5d and Supplementary Fig. 6). In order to test if the shift in EC_50_ that was caused by crowding with BSA could be a reflection of a compact conformation, this experiment additionally compared the effects on the I321E mutant that is designed to be incompetent for the compact conformation. Again, the effects on human CaMKII and its I321E mutant were indistinguishable (Fig. 5d and Supplementary Fig. 6), indicating that the observed shift in the EC_50_ did not reflect induction of a compact conformation for the full-length wild-type CaMKII. These results further confirmed that full-length CaMKII exists largely in extended conformations, even under these conditions of molecular crowding.

Finally, we tested the Ca^2+^/CaM response for a linker-less CaMKIIα mutant (LL) that was described to be in the compact conformation even without crowding. We further compared this construct to a linker-less I321E combination mutant (LL+I321E) that has been shown to remain in the extended conformation^23^. Surprisingly, without crowding, both mutants showed the same Ca^2+^/CaM sensitivity, both for activation and for T286 autophosphorylation (Fig. 5e,f and Supplementary Fig. 6). Compared with full-length CaMKII, a mild ∼1.5-fold decrease in Ca^2+^/CaM sensitivity was observed (Supplementary Fig. 6). This effect is similar to the ∼2-fold difference seen between two naturally occurring CaMKIIβ variants with different linker lengths (β and βe^31^), but not to the ∼500-fold difference previously reported for the linker-less mutant after bacterial expression^23^. Thus, after expression in mammalian cells for our kinase preparations, even the linker-less mutants were largely in an extended conformation, at least under non-crowding conditions. In contrast to full-length CaMKII, however, molecular crowding conditions differentially affected the linker-less kinase versus its I321E mutation that prevents the compact conformation. While crowding with BSA caused a shift in EC_50_ both for the linker-less kinase and its I321E mutant, this shift was significantly smaller for the I321E mutant (Fig. 5e,f). By contrast, the Hill coefficient was decreased to the same extent for both the linker-less kinase and its I321E mutant (Fig. 5e,f). These data show that the more extensive increase in the EC_50_ may provide a readout for the compact conformation for the linker-less kinase under molecular crowding conditions, while the change in Hill coefficient did not. More importantly, together these data show that after mammalian expression, crowding with BSA may induce a compact conformation for a linker-less mutant, but not for full-length CaMKII wild type. The numerical values for the effect on EC_50_ and Hill coefficient observed in our experiments are listed in (Supplementary Tables 1 and 2).

### The CaMKIIα linker promotes an extended state in cells

**Figure 6:**
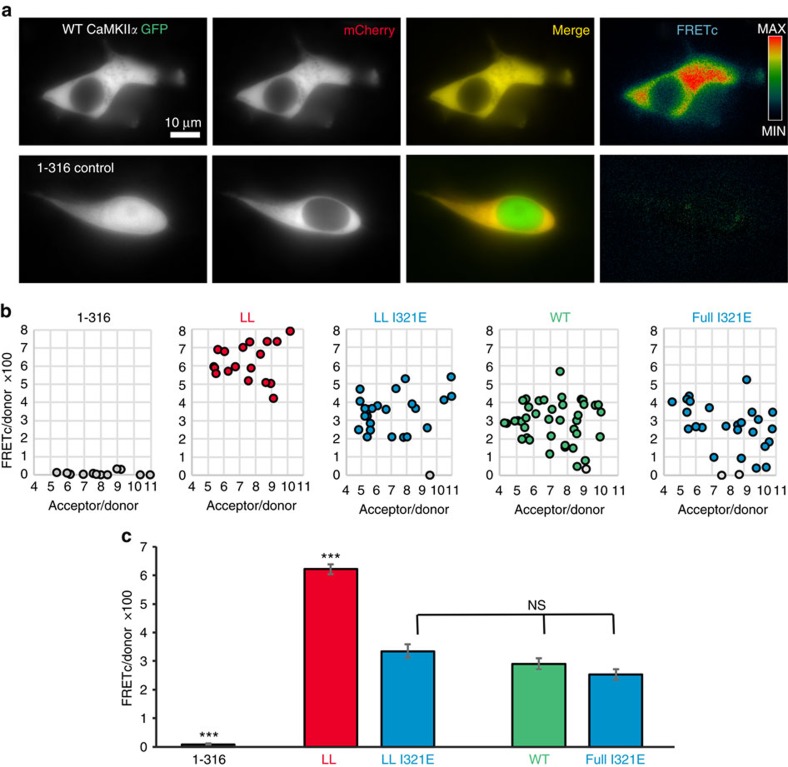
A FRET-based assay indicated a compact conformation within cells only for linker-less but not for full-length CaMKIIα wild type. (**a**) Co-expression of CaMKIIα labelled with mCherry or mGFP (as FRET donor or acceptor) N-terminal of the kinase domain resulted in a significant FRET signal in HEK cells (FRETc; corrected for fluorescence bleed-through). Deletion of the hub domain in the GFP-labelled CaMKII (1–316; *n*=12 cells) abolished the FRET signal almost completely. Scale bar=10 μm. (**b**) FRETc was normalized by expression levels of the FRET donor (FRETc/donor) and plotted as function of the acceptor/donor ratio (within a range of 4–11 fold acceptor access). The few cells that showed complete FRET failure (grey) remained included in the analysis. (**c**) The linker-less (LL) CaMKIIα mutant (*n*=17 cells) showed significantly higher FRET than all other constructs (***P<0.001; ANOVA with Tukeys post-hoc analysis). FRET of full-length CaMKIIα wild type (WT; *n*=38), of its I321E mutant (full I321E; *n*=26) that is incompetent for the compact conformation, and of a linker-less I321E mutant (LL I321E; *n*=22) were indistinguishable (NS: *P*>0.05). Error bars represent the s.e.m.

Compact versus extended CaMKII conformations result in different positioning of the kinase domains (see Fig. 5a), which would be expected to affect the FRET efficiency between labelled CaMKII subunits. Thus, we employed a FRET assay to evaluate a potential equilibrium between compact and extended CaMKII conformations by live cell imaging. Specifically, we compared full-length CaMKIIα (WT) and the linker-less mutant (LL), with and without the I321E mutation that disrupts the compact conformation. Significant FRET was detected for all of these CaMKII forms, but not for a control CaMKII (1-316) that lacks the hub (association) domain and thus cannot form multimeric holoenzymes (Fig. 6 and Supplementary Fig. 7). Note that the control CaMKII shows significant localization in both the cytoplasm and in the nucleus, consistent with its monomeric nature that abolishes the nuclear exclusion seen for the larger holoenzymes (Fig. 6a). However, this does not affect the FRET results, as the FRET measurements were restricted to the cytosol and to cells with similar cytosolic FRET acceptor (mGFP) to donor (mCherry) ratio. Specifically, an acceptor excess of 4–11-fold was selected in order to limit the analysis to cells in which the average holoenzyme (12-mer) contains at least one donor, but <3 donors (Fig. 6b). For the linker-less CaMKII, FRET was significantly reduced by the additional I321E mutation (Fig. 6c). This result is consistent with a compact conformation of the linker-less CaMKII that is disrupted by the mutation, and thus indicates that a compact conformation can be detected by this FRET assay within cells. In contrast to the linker-less mutant, full-length CaMKII wild type showed FRET that was unaffected by the I321E mutation (Fig. 6c). Thus, these data support the conclusion that full-length CaMKIIα wild type exists largely in extended conformations within cells.

## Discussion

**Figure 7:**
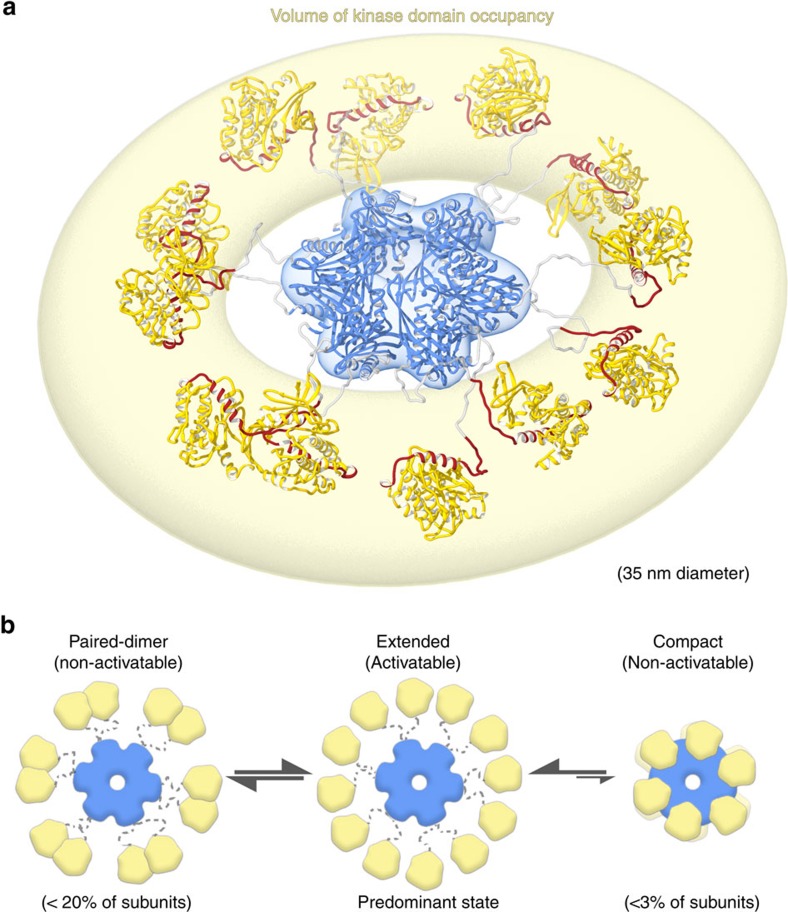
Overview of CaMKIIα kinase domain arrangements. (**a**) Illustration of the CaMKII holoenzyme indicating an overall volume of kinase domain occupancy observed by single particle EM (yellow halo). Flexible linker regions support a continuum of kinase domain arrangements within this volume of occupancy (maximum diameter of ∼35 nm). (**b**) Three major conformational states appear to exist in equilibrium. A predominant extended and activatable state, as depicted in **a**, is distinguished by an extended conformation with non-interacting kinase domains. Additional non-activatable states are distinguished by the presence of dimeric pairing between neighbouring kinase domains (representing <20% of subunits), as well as a putative compact form distinguished by kinase-hub domain interactions (representing <3% of subunits). For clarity, fully paired and fully compact states are illustrated in **b**. However, conformational states where all twelve kinase domains are simultaneously paired appear to be rare (∼2.5% of structures) and the fully compact state with all kinase subunits of an individual holoenzyme in the compact conformation was not observed at all for full-length CaMKII holoenzymes by EM or by live cell FRET analysis.

The intrinsic dynamics within the CaMKII holoenzyme architecture identified here has confounded previous attempts at obtaining a complete structural description of CaMKII. However, it is this intrinsically dynamic behaviour of CaMKII that enables the complex regulation that leads to molecular memory formation. Single particle EM is ideally suited for studying such dynamic systems because images of individual particles are obtained with extraordinarily high-resolution, and computational classification methods may effectively deconvolute inherent conformational heterogeneity. Using this approach, we have described (i) a pseudo-atomic model of the dodecameric CaMKII holoenzyme in its activation-competent extended conformation, (ii) a high flexibility of kinase domain positioning for this predominant extended state and (iii) an equilibrium with other conformational states that are activation-incompetent (Fig. 7). The predominant form is organized by the central dodecameric hub complex, with each of the twelve kinase domains tethered at peripheral positions by a flexible internal linker (the variable linker region). Two activation-incompetent states were identified as minor populations and included kinase domain dimers (<20%) and a compact conformation (<3%). An additional minor population was found with holoenzymes assembled as 14-mers (<4%) instead of 12-mers (Supplementary Fig. 3). As discussed below, these equilibria shape the regulation of CaMKII activation and may enable subunit exchange between holoenzymes.

Our 3D EM reconstruction shows a CaMKII holoenzyme in an extended activation-competent conformation at pseudo-atomic resolution, with each of the major domain components being well defined by high-resolution crystallographic structures. It should be noted that previous attempts at 3D CryoEM reconstructions have been made; however, the results were inconsistent with X-ray crystallographic structures of the organizing hub complex^32^,^33^, likely due to complications associated with particle orientation preference that were described in these studies and observed in our own work as well. For our work, complications with specimen orientation preferences were overcome by applying tomographic tilted-imaging routines, which resulted in a 3D reconstruction that matches well with individual particle images (Supplementary Fig. 5) and to previous X-ray crystallographic structures of the isolated hub and kinase domain (Fig. 2). However, while the hub domains in our reconstruction occupy a very defined volume, our results also show that the kinase domain positioning is highly flexible. Consequently, only a minor fraction of holoenzymes would be in the actual conformation of this average structure. While the average structure marks the preferred positioning for an individual kinase domain, the degree of this preference is so small that it is highly unlikely for all kinase domains of a holoenzyme to occupy this average position at the same time. As a consequence of this kinase domain flexibility, individual holoenzyme particle sizes can vary in diameter from ∼15 to 35 nm.

It was clear that kinase domain flexibility must exist in order to enable the inter-subunit autophosphorylation at T286 that generates autonomous CaMKII activity and is required for long-term synaptic plasticity. However, it was unclear if such flexibility needed to be induced by Ca^2+^/CaM stimulation, or if it is already an intrinsic property of the holoenzyme in its basal state, as shown here. The presence of such dynamic behaviour even in the basal-state suggests an additional functional role, which we propose to be involved in shaping the activation properties of the holoenzyme.

It has been previously proposed that the cooperative activation profile of CaMKII is a result of a dynamic equilibrium between an activatable extended state and a non-activatable compact state^23^. In this model, CaM binding to one subunit promotes the activatable state of neighbouring subunits, leading to cooperative activation, which in turn would shape the frequency-dependent response of CaMKII to Ca^2+^-oscillations. Indeed, we found CaMKII activation with Hill Coefficients of 1.5–2. This is consistent with previous reports, and indicates that activation by Ca^2+^/CaM is a cooperative process^20^,^32^,^34^. However, the compact conformation constituted only a very small fraction of eukaryotically expressed CaMKII holoenzymes, even under molecular crowding conditions (*in vitro* or within cells). Indication for a compact conformation was found only for a linker-less CaMKII mutant, but not for full-length (wild type) CaMKIIα holoenzymes. Thus, we conclude that cooperativity of CaMKII activation must be based on additional mechanisms.

Our data suggest cooperativity of CaMKII activation is enabled by an equilibrium with an additional activation-incompetent conformation that involves kinase domain dimerization via their Ca^2+^/CaM-binding regulatory domains. Our results indicate that such dimeric-pairs may represent as much as 20% of the total subunit population; within single holoenzymes a variety of paired and unpaired arrangements appear to be present. For isolated kinase domains, such dimerization has been captured in a crystal structure of *C. elegans* CaMKII^19^ and has been reported to occur with low affinity for human CaMKII (*K*_*d*_ 200–600 μM) (ref. 21). While this is an extremely low affinity for a specific protein–protein interaction, the local kinase domain concentration in context of the holoenzyme structure determined here is estimated to be ∼3 mM (see Methods). Under such high local concentrations, both paired and unpaired states would be significantly populated under equilibrium conditions, as observed here. In addition to affinity and concentration, the extent of kinase domain dimerization would be governed by the freedom of movement of the kinase domains, which is provided by the variable linker domain. Thus, deletion of the linker domain would be expected to impair kinase domain dimerization, which in turn should reduce cooperativity of activation. Consistent with this notion, cooperativity was found to be reduced in the linker-less CaMKII (Fig. 5 and Supplementary Tables 1 and 2). Under molecular crowding conditions, the Hill Coefficient for the linker-less CaMKII approached 1, indicating that cooperativity was almost completely abolished. Taken together, CaMKII holoenzymes exist in an equilibrium between a predominant extended, flexible, activation-competent state and two distinct and more restricted activation-incompetent states (Fig. 7). One of the activation-incompetent states is characterized by kinase domain dimerization and the other by kinase domain binding to the association domain that result in a compact conformation. However, the compact conformation constitutes only a very minor fraction and, therefore, does not significantly contribute to the cooperative CaMKII activation characteristics.

Biochemical and EM studies have established a general consensus that the CaMKII holoenzyme is formed by 12 subunits^33^,^35^,^36^. However, this was recently called into question by a study that reported a ∼1:1 ratio of 12-meric to 14-meric holoenzymes^22^. By contrast, while our study detected a small fraction of 14-meric particles (<4%), the vast majority of holoenzymes were 12-meric (Supplementary Fig. 3). Previously, it was thought that truncated association domains form 14-mers with sevenfold symmetry while full-length subunits form 12-mers with sixfold symmetry^37^. However, the majority of 14-meric particles in our study were associated with 14 kinase domains, demonstrating that 14-mers can indeed be formed by full-length subunits. By contrast, the higher extent of 14-mers in this other recent study could potentially be due to partial loss of kinase domains. Alternatively or in addition, the difference may be due to bacterial expression versus eukaryotic expression in our study. Importantly, while eukaryotic CaMKII holoenzymes are largely 12-meric, the 14-meric holoenzymes may provide a transition state that allows exchange of subunits. As stated above, it has been shown that proteolytic cleavage of the kinase domains from a 12-meric holoenzyme preparation results in the subsequent formation of 14-meric hub domain assemblies^37^. Therefore, while CaMKII wild type is exclusively observed in holoenzymes, an exchange of subunits is possible. This mechanism may allow the exchange of damaged subunits without necessitating that the entire holoenzyme be discarded. Other more speculative functions of subunit exchange have also been suggested; as such exchange can be promoted not only by kinase domain deletion, but also by stimulation^38^. These studies have used FRET-based approaches to examine subunit exchange, and the transition states are not resolved. As in the damage-induced exchange process, these transition states may be 14-meric, and our study provides direct evidence that intact full-length CaMKII holoenzyme can indeed form such 14-mers.

It remains unclear what other functional roles are enabled by the dynamic behaviour of CaMKII holoenzymes. For example, how are the various populations of conformational states affected by Ca^2+^/CaM stimulation, and what organization takes place between local kinase domains to facilitate inter-subunit trans-activation and transition to autonomously active particles. Previous FRET studies suggest additional reorganization or expansion of the holoenzyme upon stimulation by Ca^2+^/CaM^29^,^39^,^40^. This observation is consistent with our basal-state model and the expected release of the auto-inhibitory domain upon activation, which should enable a further increase the distance between the kinase and hub domains. However, it is unclear how these induced conformational changes may influence the overall dynamic behaviour of the signalling particle. Furthermore, it is uncertain how the holoenzyme architecture enhances binding to GluN2B, enabling molecular memory formation in this context^6^,^9^,^10^. Future experiments aimed at addressing these questions are almost certain to provide new insights into the enigmatic mechanism of molecular memory formation, and unveil more surprising features of this remarkable signalling complex.

## Methods

### CaMKII and CaM preparations

Rat CaMKIIα wild type was purified after baculovirus/S*f*9 cell expression and CaM was purified after bacterial expression. Briefly, CaM was purified by differential ammonium sulfate precipitation followed by phenyl-sepharose columns^41^. CaMKII was purified from cytoplasmic 100,000*g* supernatants via two sequential column purification steps, a P11 phospho-cellulose column followed by a CaM-sepharose affinity column^42^,^43^.

Human CaMKIIα and CaMKIIα mutants were expressed in HEK-293 cells. The cells were grown to ∼50% confluence in 10 cm dishes, then transfected using the Ca^2+^-phosphate method^44^,^45^. After two days of expression, HEK cells were homogenized with a motorized pellet pestle (Kontes) for 10 s in 0.4 ml of ice-cold 50 mM PIPES pH 7.2, 10% glycerol, 1 mM EDTA, 1 mM dithiothreitol, and complete protease inhibitor (Roche), then centrifuged at 16,000*g* for 20 min. The CaMKII concentration in the resulting supernatant was determined by quantitative western blot using a standard curve containing 250–1,250 fmole of purified recombinant CaMKIIα diluted in non-transfected HEK cell extract. Blots were probed for CaMKIIα expression using CBα2 antibody (1:2,000, produced in-house).

### CaMKII activity assay

CaMKII activity was measured by ^32^P incorporation into the peptide substrate Syntide-2. Reactions were started by adding purified CaMKIIα or HEK cell extract containing CaMKIIα to a final concentration of 2.5 nM in a mix of 50 mM PIPES pH 7.2, 0.1% BSA, 2 mM CaCl_2_, 10 mM MgCl_2_, 100 mM [γ-^32^P]ATP (∼1 Ci mmole^−1^) 1 μM microcystine-LR, 75 mM Syntide-2 and 3 nM to 20 μM calmodulin^42^,^43^. Some reactions additionally contained 150 mg ml^−1^ BSA or lysozyme, as indicated. Mixtures (50 μl) were reacted for 3 min at 30 °C. Reactions were stopped by adding 15 μl of ice cold 15% TCA, vortexing, and incubating on ice for 20 min. Reactions were then centrifuged at 16,000*g* for 20 min to remove precipitated protein. Thirty-five microlitres of the supernatant containing the peptide substrate was spotted onto P81 cation exchange chromatography paper (Whatman) squares. After extensive washes with water, phosphorylation of the substrate peptide bound to the P81 paper was measured by liquid scintillation counting. Results were plotted using Graph Pad Prism 5 and fit using a non-linear regression with variable slope.

### CaMKII autophosphorylation assay

To assess the ability of CaMKIIα WT and mutants to autophosphorylate at T286, the kinases were added at a final concentration of 20 nM to a reaction buffer containing 50 mM PIPES pH 7.2, 0.1% BSA, 2 mM CaCl_2_, 10 mM MgCl_2_, 100 mM ATP, 1 μM microcystin-LR, 100 nM calmodulin. Kinases were reacted at 30 °C for 30 seconds, or as indicated. Reactions were stopped by addition of gel loading buffer (2% SDS, 50 mM dithiothreitol, 67.5 mM Tris pH 6.8, 10% glycerol, 0.16 mg ml^−1^ bromophenol blue) and boiling for 5 min. Samples were loaded on 10% SDS–polyacrylamide gel electrophoresis gels then transferred to polyvinylidene difluoride membranes^42^,^43^. Blots were blocked in 5% milk then probed with anti-phospho-T286 CaMKII antibody (Phosphosoutions) diluted 1:3,000 in 1% milk. Images were acquired on an Alpha Imager (Alpha Innotech) after exposure to Western Lightning ECL reagent (Perkin Elmer) and quantified^42^,^43^,^44^.

### FRET microscopy and image analysis

HEK-293 cells (authenticated by short tandem repeat (STR) analysis and tested for mycoplams) were grown to 50% confluency on glass coverslips and then transfected using the Ca^2+^/phosphate method, using a 4:1 ratio of mCherry over green fluorescent protein (GFP) constructs. Twenty-four hours after transfection, images of live cells were acquired at 32 °C on a climate controlled Zeiss Axiovert 200M microscope (Carl Zeiss GmbH, Oberkochen, Germany) at 100 × magnification using slide book 5.5 software (Intelligent Imaging Innovations) in imaging solution containing 0.87 X HBSS, 25 mM HEPES, 2 mM Glucose, 2 mM CaCl_2_, 1 mM MgCl_2_. FRET image acquisition and analysis were done by the three-filter ‘micro-FRET’ image subtraction method^46^. In brief, three single plane images (40-ms to 500-ms exposure sets, 2 × 2 binning) were obtained: a GFP excitation/GFP emission image; a mCherry excitation/mCherry emission image; and a GFP excitation/mCherry emission image (raw, uncorrected FRET). Background-subtracted GFP and mCherry images were then fractionally subtracted from raw FRET images based on measurements for GFP bleedthrough (0.02093 fraction of GFP image) and mCherry cross-excitation (0.09484 fraction of mCherry image). This fractional subtraction generated corrected FRET_C_ images, represented in pseudo-colour. The fractional subtraction coefficients are rounded up from average cross-bleed values determined in cells expressing GFP- or mCherry-tagged constructs alone. Thus, these coefficients result in a slight underestimation of FRET_C_ signals for true FRET partners but limit false positive detection of FRET.

Absolute FRET_C_ values depend both on FRET efficiency and on the amount of fluorophores present. Thus, in order to compare FRET among different cells, the FRET_C_ values were divided by the intensity of the donor fluorophore. This simple normalization method is valid when the acceptor is in excess; here, only cells with 4–11-fold acceptor excess were included in the analysis. Acceptor/donor ratio was determined based on the detected GFP/mCherry signal combined with the 4.8 more efficient fluorescence detection of GFP in our setup (as determined in cells expressing a GFP-mCherry fusion protein).

Image acquisition was done based on the GFP and mCherry channels, that is, blind of the raw FRET channel. Image analysis was performed blind of the condition.

### CaMKII preparation for EM

Full-length CaMKIIα purified from eukaryotic S*f*9 cell expression was prepared for negative stain EM by diluting the purified protein (1:40 vol vol^−1^) in EM buffer containing 50 mM HEPES (pH 7.4), 120 mM KCl and 0.5 mM EGTA. A 3 μl drop of sample (∼100 nM) was applied to a glow-discharged continuous carbon coated EM specimen grid (Ted Pella). Excess protein was removed by blotting with filter paper, and washing the grid two times with EM buffer. The specimen was then stained with freshly prepared 0.75% (wt vol^−1^) uranyl formate (SPI-Chem). Cryogenically prepared specimens were prepared by applying a 3 μl drop of sample (∼1 μM) to a negatively charged Quantifoil holey carbon specimen grid (Electron Microscopy Science). The sample was blotted with filter paper and plunged into liquid ethane using a vitrobot (FEI) and stored under liquid nitrogen.

### EM and image processing

Negatively stained specimens were visualized on a 120 kV TEM (iCorr, FEI) at a nominal magnification of 49,000 × at the specimen level. Digital micrographs were recorded on a 2 K × 2 K CCD camera (FEI Eagle) with a calibrated pixel size of 4.37 Å pixel^−1^ and a defocus of 1.5–2.5 μm. To overcome issues with particle orientation preference on the EM grid, serial tomographic images were collected at tilts angles of 0°–50° (Δ 10°) (Supplementary Fig. 5)^47^. Contrast transfer function (CTF) parameters were determined in EMAN2 (ref. 48) and micrographs free of significant astigmatism and drift were selected based on Thon rings in the power spectra. A total of 16,616 particles were hand selected in EMAN2 (10902 un-tilted and 5,714 tilted particles) and extracted with a box size of 128 × 128 pixels. Reference-free 2D class averages were generated in EMAN2 (ref. 48) and RELION v1.4 (ref. 49) using CTF-corrected (phase-flipped) and band-pass filtered images without any applied symmetry. 2D variance maps were calculated in RELION by squaring the s.d. of aligned particle images present in the 2D class average.

To separate coexisting conformational states of the CaMKIIα hub domain (dodecamer or tetradecamer), a focused reference-free 2D classification was preformed in RELION using a subset of ∼8,000 un-tilted particles with a soft outer mask (75 Å radius) applied before classification. A set of 80 classes was produced and analysed to determine the relative populations of hub domains comprising of sixfold and sevenfold symmetry (Supplementary Fig. 3). The constituent particles from classes with apparent sevenfold symmetry were extracted as unmasked images, and reclassified for validation. For analysis of the kinase domains, a soft inner mask (50 Å radius) was applied to remove densities corresponding to the hub domain before 2D classification.

Cryogenically prepared CaMKIIα particles were imaged on a 300kV Titan Krios (FEI). Digital micrographs were recorded on a Falcon II direct electron detector (FEI) using low-dose imaging routines at a nominal magnification of 47,000 × and defocus of 3–5 μm. CTF correction and image processing routines were carried out in EMAN2, as described above. 2D class averages were obtained from a small dataset of ∼500 individual particle images.

### Single particle measurements and statistical analysis

Statistical analyses of individual particle dimensions were obtained by measuring particle lengths on un-binned micrographs using the measurement tool in EMAN2. A random set of 82 representative un-tilted individual particle images was inspected. A radius of extension for individual kinase subunits (*n*=928) was obtained by measuring the distance from the center of the pore in the hub domain complex to the center of each peripheral density corresponding to the kinase domains. For each of these measurements, a distance of 22.5 Å was appended (corresponding to the average radius of the kinase domain) to yield a value that represents the full extension of the kinase domain. Inter-kinase domain separation distances were determined by measuring from the center of one peripheral kinase domain density to the center of the closest neighbouring density in the clockwise direction. Only densities that could be clearly resolved as individual kinase domains were incorporated into this analysis (*n*=883). The distribution of individual particle measurements were binned into 5 Å increments for histogram analysis (corresponding approximately to the pixel value of these images=4.37 Å). A standard Gaussian curve was fit to the distribution using the experimentally determined mean and s.d. Whisker plots were generated to represent the minimum, maximum and 25%, 50%, and 75% quartile values for each data set. A correlation map was obtained by scatter plot analysis of kinase radius versus kinase domain separation. All statistical analyses were performed in Microsoft Excel.

### 3D reconstruction and refinement

An initial 3D reconstruction was determined using EMAN2 from a culled subset of tilted and un-tilted particles showing clearly defined hub domains and separated kinase domains in 2D class averages. A calculated map for the isolated human CaMKIIα hub domain (PDBID 51G3 (ref. 22)) was filtered to 40 Å and used as a search model for initial alignments. This produced a ∼25 Å resolution reconstruction with imposed D6 symmetry displaying strong density for the central hub domain and weak peripheral densities representing the twelve kinase domains assembled by the dodecameric holoenzyme (Supplementary Fig. 4). This initial reconstruction was filtered to 60 Å and used as a reference for masked 3D refinements in RELION.

Separate refinements of the hub domain and kinase domains were performed in RELION by incorporating a 3D Gaussian mask based on the central hub feature produced by the initial 3D reconstruction in EMAN (Supplementary Fig. 4). For the hub-only refinement, the mask was set to remove peripheral densities corresponding to the kinase domains during the alignment procedure (_max_diameter=∼125 Å). For the kinase domain refinement, the 3D mask was extended by 22 Å and inverted to remove density corresponding to the central hub domain during alignment. Both refinements proceeded with applied D6 symmetry. The hub-only refinement was determined to a resolution of ∼19 Å (gold standard FSC). For the kinase domain refinement, a subset of images was obtained from a combination of 2D and 3D classification performed in RELION. For 3D classification, six classes were generated with D6 symmetry imposed. Four of the six classes had clearly resolved kinase domains and the particles in these classes were selected for further refinement, resulting in a final resolution of ∼20 Å (gold standard FSC). These two maps were then combined using a spherical mask to remove overlapping densities (radius=75 Å). A final combined density map representing the full-length holoenzyme has been deposited to the EM Data Bank (EMD-8514).

### Molecular modelling of the human CaMKIIα holoenzyme

A pseudo-atomic model of the full-length CaMKIIα holoenzyme was constructed by fitting the atomic coordinates of the dodecameric human CaMKIIα hub domain (PDBID 5IG3 (ref. 22) residues 345–472) and monomeric human CaMKIIα kinase/regulatory domain (PDBID 2VZ6 (ref. 21) residues 13–300) into the EM density map by rigid body fitting using UCSF Chimera^50^. The orientations of the kinase domains were chosen based on best fit to the map. Two neighbouring kinase domains were fit separately and symmetrized to reflect the sixfold dihedral symmetry in the EM map. The fitted domains gave good agreement to the experimental density (cross-correlation at 20 Å resolution=0.95 for the hub complex and 0.98 for kinase domains). A flexible linker connecting these two structured domains (residues 301–344, human sequence), including the distal region of the CaM-binding site (residues 301–314) and variable linker region (residues 315–344). Linkers were constructed separately for each chain using the MODELER loop building tool^25^, and subjected to steepest descent minimization and subsequent conjugate gradient minimization routines in Chimera to regularize the geometries and remove steric interactions. Atomic coordinates for the constructed model have been deposited with the Protein Data Bank (PDBID 5U6Y).

A model for calculating the local concentration of kinase domains within the CaMKII holoenzyme was based on calculating the volume of a torus (Volume=(*πr*^2^) × (2*π*R)); where *R* and *r* represent the major and minor radius of the torus, respectively), as defined by the minimum and maximum kinase radius of extension determined in this work (providing *R*=126 Å and *r*=49 Å). This volume, representing the space occupied by kinase domains surrounding the central hub complex, was then used to estimate the local concentration of kinase domains for a dodecameric holoenzyme, resulting in ∼3.3 mM, which corresponds well to previously determined estimates^22^.

Protein structures were visualized and images captured in UCSF Chimera. Figures were prepared in Adobe Photoshop. The contrast of EM micrographs and 2D class averages were similarly adjusted for manuscript presentation.

### Data availability

The EM map has been deposited with the EM database (EMD-8514). The pseudo-atomic model of the CaMKII holoenzyme fit to the EM density has been deposited with the Protein Data Bank (PDBID 5U6Y). The data that support the findings of this study are available from the corresponding authors upon request.

## Additional information

**How to cite this article:** Myers, J. B. *et al*. The CaMKII holoenzyme structure in activation-competent conformations. *Nat. Commun.* **8,** 15742 doi: 10.1038/ncomms15742 (2017).

**Publisher’s note**: Springer Nature remains neutral with regard to jurisdictional claims in published maps and institutional affiliations.

## Supplementary Material

Supplementary Information Supplementary Figures, Supplementary Tables and Supplementary References

Supplementary Movie 1 3D reconstruction and pseudo-atomic modeling of the CaMKIIα holoenzyme.
