# Publisher Correction: The CaMKII holoenzyme structure in activation-competent conformations

**DOI:** 10.1038/ncomms16180

**Published:** 2018-05-25

**Authors:** Janette B. Myers, Vincent Zaegel, Steven J. Coultrap, Adam P. Miller, K. Ulrich Bayer, Steve L. Reichow

Nature Communications 8: Article number: 15742; DOI: 10.1038/ncomms15742 (2017); Published 06 07 2017; Updated 05 25 2018

The previously published version of this Article contained an error in Figure 7. In panel b, the % Compact subunits was shown as ‘<30%’ but should have instead been ‘<3%’. This error has been corrected in both the PDF and HTML versions of the Article.

