# Supplementary material for: The CaMKII holoenzyme structure in activation-competent conformations
Source: Nat Commun. 2017 Jun 7;8:15742. doi: 10.1038/ncomms15742 (PMC5467236; doi:10.1038/ncomms15742)
Supplement: Supplementary Information — Supplementary Figures, Supplementary Tables and Supplementary References [file ncomms15742-s1.pdf]

Supplementary Figures and Legends

Supplementary Figure 1

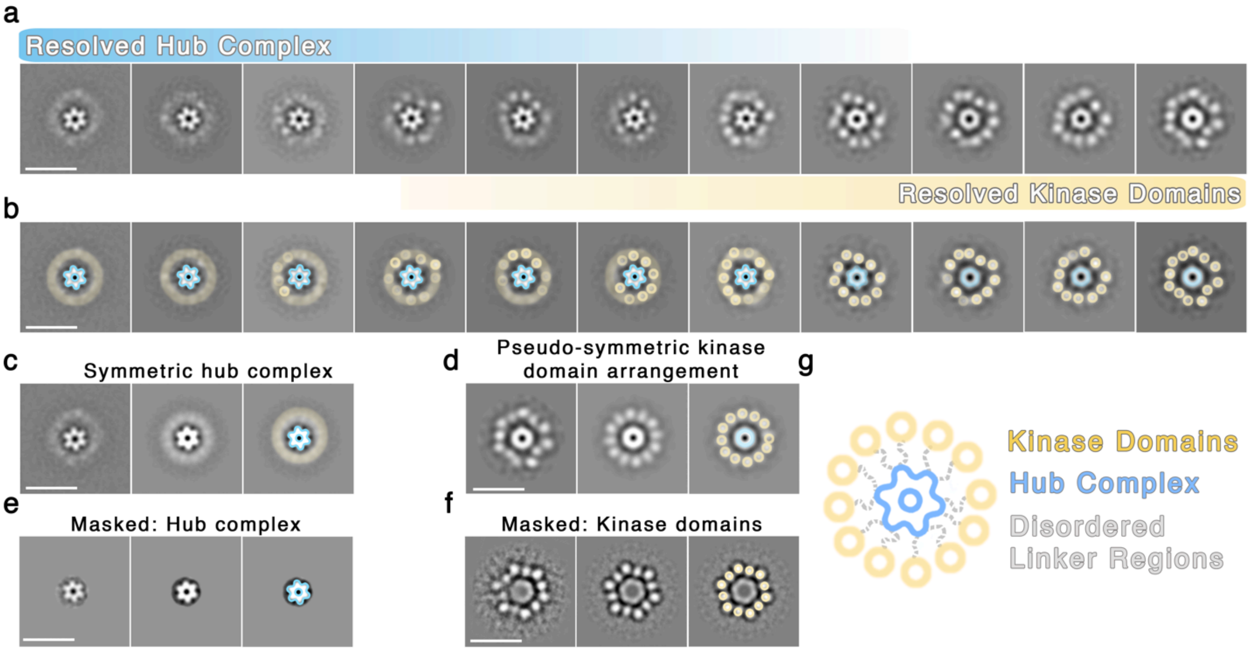

Supplementary Figure 1: Overview of 2D classification routines and analysis. (a) Reference-free 2D class averages obtained from unmasked images result in a continuum of structures, displaying either a well-resolved hub complex *(left)* or up to twelve resolved kinase domains *(right)*. Scale bar in all panels = 25 nm. **(b)** Same as panel **a**, with resolved hub complex *(blue outline)* and kinase domains *(yellow circles)* indicated. Faded circles *(yellow and blue)* indicate regions of unresolved structure. **(c)** Representative class average with a well-resolved hub complex *(left)*, and with applied 6-fold rotational symmetry *(middle and right)*. **(d)** Representative class average with twelve resolved kinase domains *(left)*, with applied 6-fold rotational symmetry *(middle and right)*. **(e)** Representative class average obtained with an applied 75 Å outer radius mask to remove contributions of the kinase domain during the alignment procedure *(left)*, and with applied 6-fold rotational symmetry *(middle and right)*. **(f)** Representative class average obtained from particle images with an applied 50 Å inner radius mask to remove contributions of the hub domain *(left)*, and with applied 6-fold rotational symmetry *(middle and right)*. Symmetry was only applied after 2D classification results. **(g)** Illustration of domain positions observed in symmetrized class averages from panels **e** and **f**.

Supplementary Figure 2

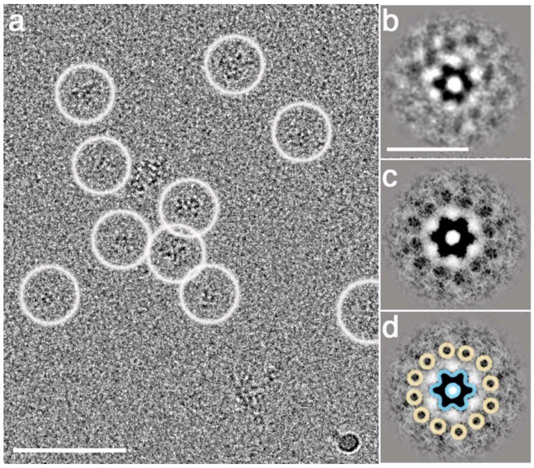

Supplementary Figure 2: Vitrified CaMKIIα particles imaged by electron cryo-microscopy. **(a)** Electron micrograph obtained from cryogenically prepared CaMKIIα holoenzymes *(contrast of protein is dark on lighter background)*. Isolated single particles are indicated by white circles. Scale bar = 100 nm. **(b)** 2D class average obtained from a small test dataset of ~500 particles indicates that frozen hydrated CaMKIIα particles adopt structures similar to those observed by negative stain EM. Scale bar = 25 nm. **(c, d)** Class average in panel **b** with applied 6-fold rotational symmetry enhances the contrast and pseudo-symmetric arrangement of up to twelve kinase domain densities and the 6-fold symmetric hub domain complex *(yellow circles and blue outline in panel* ***d****, respectively)*.

Supplementary Figure 3

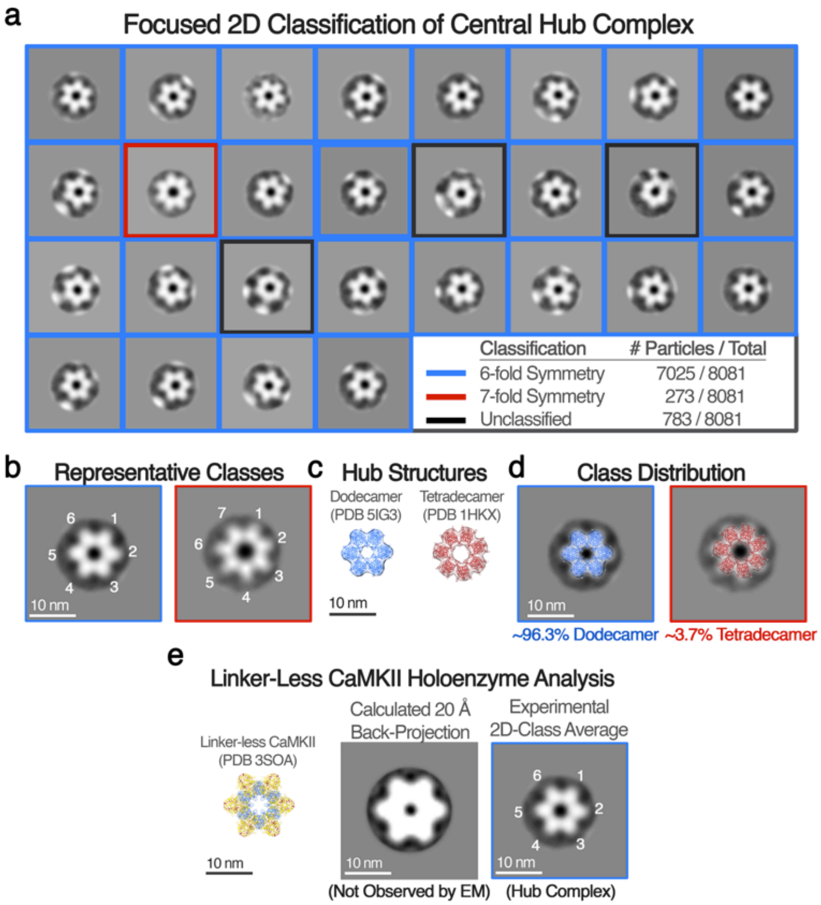

Supplementary Figure 3: Focused 2D classification of hub complex structures observed by negative stain EM. (a) Focused 2D classification results of the hub domain complex were obtained using an image mask (75 Å outer radius) that was applied to remove contributions of the kinase domains during the alignment procedure (28 of a total 80 classes are displayed). No symmetry was applied during classification and alignment. Class averages were categorized as displaying 6-fold symmetry *(blue outline)*, 7-fold symmetry *(red outline)*, or unclassified *(black outline)*. *Inset,* Indicates the population of particles belonging to each category. **(b)** Representative class average of hub domain complexes with identified 6-fold symmetry *(left)* and 7-fold symmetry *(right)*. Scale bar in all panels = 10 nm. **(c)** Crystallographic structures reported for dodecameric (*blue ribbon*, PDBID 5IG3[1]) and tetradecameric (*red ribbon*, PDBID 1HKX[2]) assemblies of the isolated hub complex. **(d)** Crystal structures in panel **c** fit into the EM densities in panel **b**. The two models fit with good agreement to their respective 2D class averages. The percentage of classified hub complexes identified as dodecameric and tetradecameric were 96.3% and 3.7%, respectively. **(e)** Ribbon representation of the linker-less (LL) CaMKII crystal structure (PDBID 3SOA)[3] *(left)*, corresponding back-projection calculated at

20 Å *(middle)*, and representative class-average of the hub complex *(right)*. The overall shape of the back-projected crystal structure looks similar to the 2D class average of the hub-complex, but with an increased radius of ~100 Å *(compare middle and right)*. Focused 2D classification (mask = 110 Å outer radius) does not identify any structures corresponding to the dimensions of the crystallized (LL) CaMKII holoenzyme in our dataset.

Supplementary Figure 4

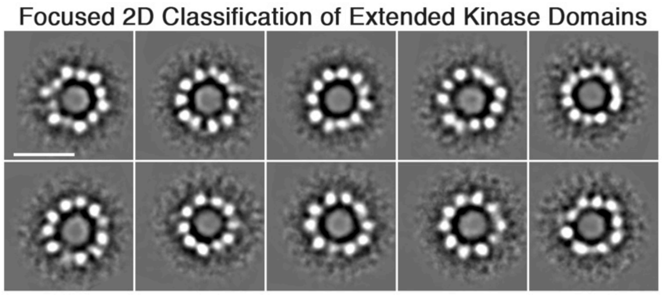

Supplementary Figure 4: Focused 2D classification of kinase domain arrangements. A set of representative 2D class averages obtained from negatively stained CaMKII particle images using an applied inner mask (inner diameter = 50 Å) to remove contributions from the central hub feature. Scale bar = 25 nm. The resulting projection averages display clearly resolved kinase domain densities arranged along a radius of ~24 – 28 nm. These most populated classes reveal 9 – 12 resolved densities.

Supplementary Figure 5

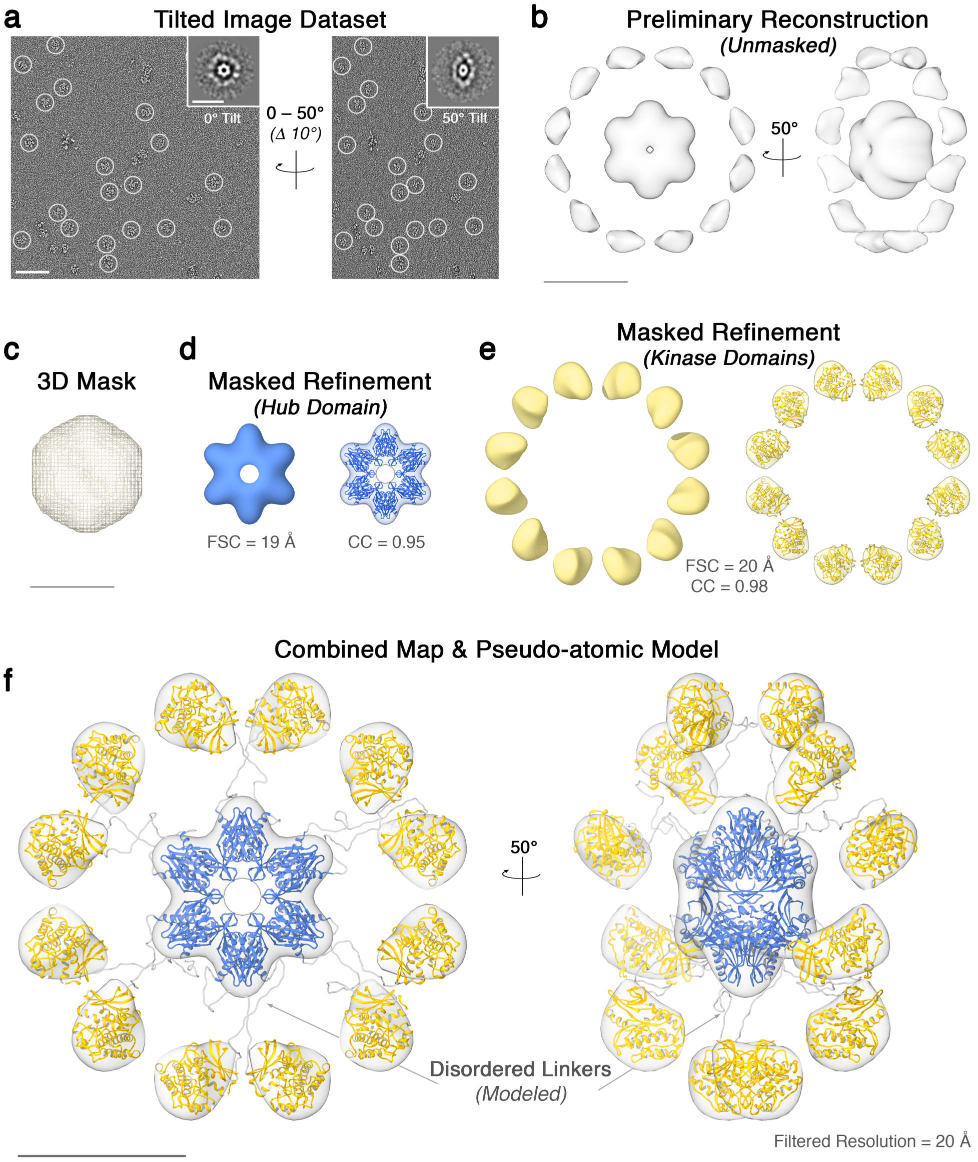

Supplementary Figure 5: Workflow of 3D reconstruction and pseudo-atomic modeling of the CaMKIIα holoenzyme. (a) Representative micrographs of negatively stained CaMKIIα particles recorded with the specimen stage tilted at 0° *(left)* and 50° *(right)*. Individual particles are indicated by white circles. A complete image dataset of ~16,000 particles were collected at varying stage tilts of 0°, 10°, 20°, 30°, 40° and 50°. Scale bar = 100 nm. *Insets,* show representative reference-free 2D class averages. Scale bar = 25nm. **(b)** Initial 3D reconstruction obtained from unmasked particles using EMAN2[4]. Scale bar = 100 Å. **(c – e)** Masked 3D refinement strategy. Scale bar = 100 Å. **(c)** 3D mask used for refinements of the central hub complex and peripheral kinase domains performed in RELION v1.4[5]. **(d)** EM map of the hub

domain complex obtained using the 3D mask in panel **c**. The map was refined to 19 Å (gold standard FSC) with applied 6-fold dihedral symmetry as defined by the hub complex. The crystal structure of the human dodecameric CaMKIIα hub complex (*blue ribbon,* residues 345 – 472; PDBID 5IG3[1]) fit well with the EM density map (correlation coefficient = 0.95 at 20 Å). **(e)** EM map of the CaMKIIα kinase domains obtained using an inverted 3D mask shown in panel **c**. The map was refined to 20 Å (gold standard FSC) with applied 6-fold dihedral symmetry. The crystal structure of the isolated human CaMKIIα kinase/regulatory domain (*yellow ribbon,* residues 13 – 300; PDBID 2VZ6[6]) fit well within each of the twelve EM densities (correlation coefficient = 0.98 at 20 Å). **(f)** A final EM map was constructed by merging EM densities shown in panels **d** and **e**. A pseudo-atomic model of the CaMKIIα holoenzyme was completed by connecting the crystal structures fit into the EM density map by random coil chains. Each kinase/regulatory domain (residues 13 – 300) is connected to the nearest hub domain (residues 345 – 472) using a disordered linker (grey coil, residues 301 – 344). The disordered linker for each peptide chain was calculated separately using MODELER[7]. Scale bar = 100 Å.

Supplementary Figure 6

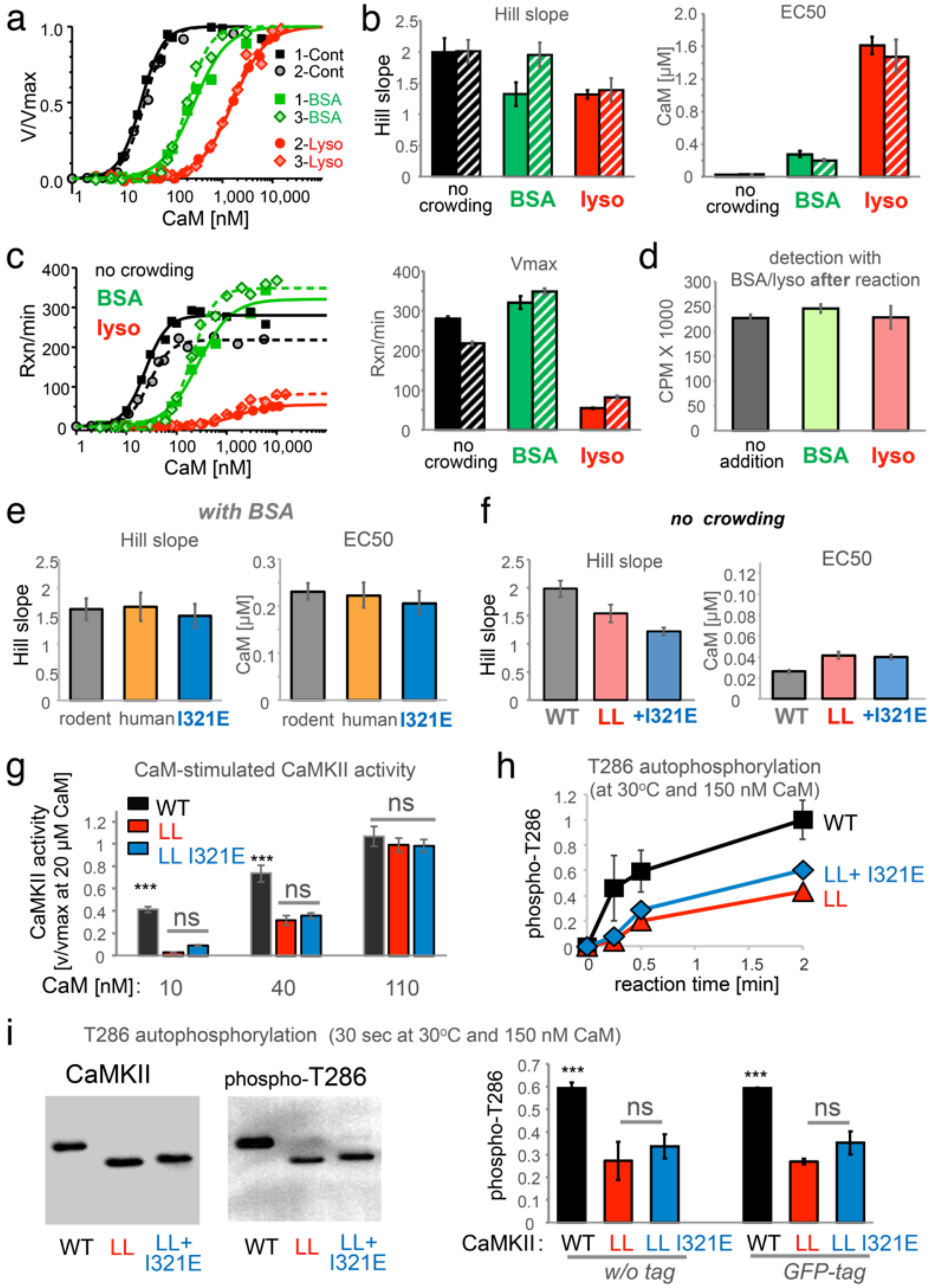

Supplementary Figure 6: CaMKIIα activation by Ca^{2+}/CaM *in vitro* and the effects of molecular crowding (by 150 mg/ml BSA or lysozyme), a linker-less deletion mutant (LL; described to favor the compact conformation), and an I321E mutant (described to prevent the compact conformation). (a, b) Ca^{2+}/CaM dose/response curves for *in vitro* activation of rodent CaMKII without crowding (Contr) or with crowding by 150 mg/ml BSA or

lysozyme (lys) was determined in two independent experiments for each condition (with two conditions tested in parallel in each of three experiments, as indicated). While **Fig. 5b,c** show fits for the combined data points, here, curve fits and analysis are shown separately for each experiment. While the Hill slope is a sensitive measure, any indication for variability was seen only for BSA. Importantly, no increase in the Hill slope by molecular crowding was detected in any of the individual experiments. **(c)** Dose response as in panel **a**, but plotted as absolute kinase activity (in reactions per minute) instead of activity normalized to V_{max}. This indicated that V_{max} was slightly increased by crowding with BSA, but strongly decreased by lysozyme. **(d)** Addition of BSA or lysozyme after the kinase reaction (but before detection of the phosphorylated substrate) did not affect detection of the phosphorylated substrate. Thus, lysozyme affected the V_{max} in the kinase reaction, not the detection after the reaction. **(e)** Rodent, human, and human I321E mutant CaMKIIα showed identical Hill slope and EC_{50} during molecular crowding with BSA. Shown is the analysis of the dose response curves in **Fig. 5d**. **(f)** Without crowding, the linker-less mutant (LL) did not show any increase in Hill slope, thus indicating no compact conformation, at least when expressed in mammalian cells. The EC_{50} of the linker-less mutant showed only a very mild ~1.5 fold increase, and this was identical for the linker-less I321E mutant. Thus, the linker-less mutant was not in a compact conformation without molecular crowding; however, with molecular crowding, a compact conformation could be induced (see **Fig. 5e, f**). **(g)** An independent experiment verified identical Ca^{2+}/CaM dose response for the linker-less mutant and its I321E mutant without molecular crowding. Compared to full-length CaMKIIα wild type, a reduced response was seen at lower CaM concentrations (10 and 40 nM), but not at higher CaM concentration (100 nM), consistent with a rather mild shift in Ca^{2+}/CaM-sensitivity. Kinase activity was normalized to maximal activity induced with 20 μM CaM. **(h)** Time course of CaMKII T286 autophosphorylation at 30° C with 1 mM Ca^{2+} and 150 nM CaM. Autophosphorylation was detected by Western blot analysis and quantified as described (see also panel **i**). **(i)** Compared to CaMKIIα wild type, T286 auto-phosphorylation was reduced to the same extent for linker-less CaMKII and its I321E mutant. The same result was obtained for GFP-tagged versions of the kinase. The autophosphorylation conditions chosen (30 sec at 30° C with 150 nM CaM) induced sub-maximal phosphorylation for CaMKII wild type (here ~60% compared to extended 2 min reactions; see panel **h**). Autophosphorylation was detected by Western blot analysis *(left)* and quantified *(right)* as described. Error bars in panels b-f represent the calculated standard error from the curve fits; error bars in panels g-i represent the standard error of mean (s.e.m.).

Supplementary Figure 7

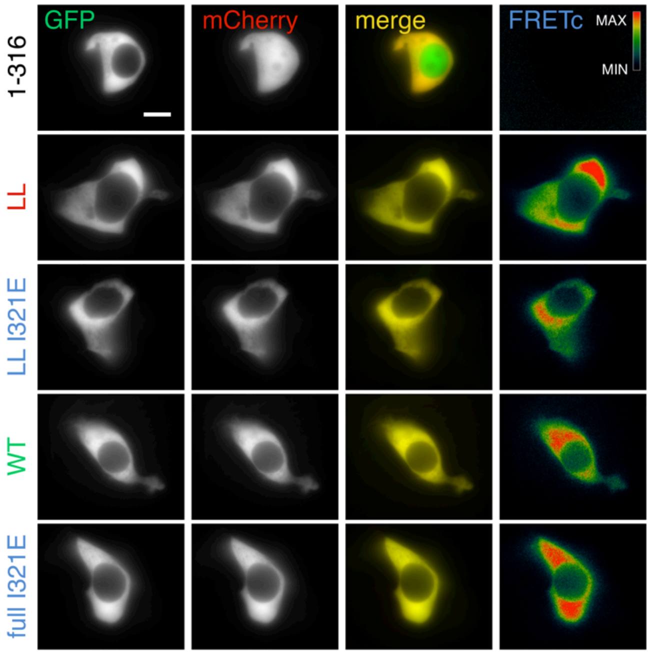

Supplementary Figure 7: A FRET-based assay indicated a compact conformation within cells only for linker-less but not for full-length CaMKIIα wild type. Shown here are example images for all conditions quantified in **Fig. 6** of main text. This includes a truncated CaMKII control without the association domain (1-316), the linker-less kinase (LL), the linker-less kinase with the I321E mutation described to disrupt the compact conformation (LL I321E), full-length wild type kinase (WT), and full-length kinase with the I321E mutation (full I321E). Scale bar = 10 μm.

Supplementary Tables and Legends

CaMKIIα	exp.	*EC₅₀ [nM]*		*Hill Coefficient*	
		Without BSA	With BSA	Without BSA	With BSA
rodent	1	23.8 +1.58/-1.48	276.7 +43.0/-37.21	2.00 +/-0.227	1.32 +/-0.192
	2	28.4 +1.66/-1.57	203.2 +13.0/-12.21	2.08 +/-0.182	1.95 +/-0.192
	3	nd	231.2 +17.7/-16.42	nd	1.62 +/-0.189
human		nd	222.3 +27.8/-24.68	nd	1.66 +/-0.253
human I321E		nd	206.0 +26.2/-23.22	nd	1.50 +/-0.210

Supplementary Table 1. Molecular crowding with 150 mg/ml BSA does not increase the Hill coefficient for mammalian-expressed CaMKIIa. If any, a slight decrease is observed. The significant increase in EC_{50} caused by molecular crowding is not related to induction of the compact conformation (at least for the full-length kinase tested in this Table), as it is indistinguishable for the I321E mutant that is incompetent for the compact conformation. Effect on rodent versus human CaMKII was also indistinguishable.

CaMKIIα	exp.	*EC₅₀ [nM]*		*Hill Coefficient*	
		Without BSA	With BSA	Without BSA	With BSA
LL	1	42 +3.4/-3.2	2570 +668/-530	1.54 +/-0.156	0.99 +/-0.091
	2	nd	1841 +655/-483	nd	1.10 +/-0.185
LL + I321E	1	40 +2.3/-2.2	849 +159/-133	1.22 +/-0.070	1.06 +/-0.119
	2	nd	1099 +106/-96	nd	1.18 +/-0.083

Supplementary Table 2. Molecular crowding with 150 mg/ml BSA might induce a compact conformation for linker-less (LL) CaMKII. However, the differential effect of crowding on the I321E mutant that is incompetent for the compact conformation manifested in EC_{50} rather than the Hill coefficient.

Supplementary References

1. Bhattacharyya, M. et al. Molecular mechanism of activation-triggered subunit exchange in Ca(2+)/calmodulin-dependent protein kinase II. *Elife* **5**(2016).
2. Hoelz, A., Nairn, A.C. & Kuriyan, J. Crystal structure of a tetradecameric assembly of the association domain of Ca2+/calmodulin-dependent kinase II. *Mol Cell* **11**, 1241-51 (2003).
3. Chao, L.H. et al. A mechanism for tunable autoinhibition in the structure of a human Ca2+/calmodulin- dependent kinase II holoenzyme. *Cell* **146**, 732-45 (2011).
4. Tang, G. et al. EMAN2: an extensible image processing suite for electron microscopy. *J Struct Biol* **157**, 38-46 (2007).
5. Scheres, S.H. RELION: implementation of a Bayesian approach to cryo-EM structure determination. *J Struct Biol* **180**, 519-30 (2012).
6. Rellos, P. et al. Structure of the CaMKIIdelta/calmodulin complex reveals the molecular mechanism of CaMKII kinase activation. *PLoS Biol* **8**, e1000426 (2010).
7. Fiser, A., Do, R.K. & Sali, A. Modeling of loops in protein structures. *Protein Sci* **9**, 1753-73 (2000).
